# Ross River Virus Risk Associated with Dispersal of *Aedes (Ochlerotatus) camptorhynchus* (Thomson) from Breeding Habitat into Surrounding Residential Areas: Muddy Lakes, Western Australia

**DOI:** 10.4269/ajtmh.13-0399

**Published:** 2014-07-02

**Authors:** Andrew Jardine, Peter J. Neville, Colin Dent, Carla Webster, Michael D. A. Lindsay

**Affiliations:** Mosquito-Borne Disease Control, Environmental Health Hazards Unit, Environmental Health Directorate, Department of Health Western Australia, Perth, Western Australia, Australia; Health Services, Shire of Capel, Capel, Western Australia, Australia

## Abstract

Rapid population growth in Western Australia has resulted in increased development of land for residential housing, and new developments are often proposed close to water because of intrinsic aesthetic values. However, this placement may place future residents at risk of mosquito-borne disease, of which Ross River virus (RRV) disease is the most common in Australia. Mosquito dispersal data were combined with a spatial analysis of human RRV cases to show that mosquitoes dispersed readily from larval habitat into surrounding low- and high-density residential areas and that residents living within 2 km of mosquito breeding habitat had a significantly higher rate of RRV disease. This finding highlights the importance of planning authorities in state and local governments to consider the implications of mosquito-borne disease risks when assessing residential development applications.

## Introduction

Many residential areas in southwestern Western Australia (WA) are located in close proximity to major natural mosquito breeding habitat, and many more are being developed because of aesthetic values of living near water. However, residents in such areas may be exposed to greater risk of contracting mosquito-borne diseases, such as Ross River virus (RRV) (Togaviridae: Alphavirus), and intense nuisance problems at certain times of year when mosquitoes disperse from breeding habitat into surrounding suburbs. Rapid population growth and subsequent pressure to develop new areas for residential housing are further exacerbating the problem.

RRV disease is the most common mosquito-borne disease in Australia, causing a non-fatal but potentially debilitating polyarthritic disease in humans, with approximately 5,000 human cases notified annually to health departments across Australia.^1^ In southern coastal areas of WA, disease outbreaks often occur when rainfall and tides enable mosquito vector populations, particularly *Aedes (Ochleratatus) camptorhynchus* (Thomson), to persist into warmer months.^2^,^3^ This species primarily breeds in coastal salt marshes^4^ but has also been collected in moderate numbers in brackish inland waters.^5^,^6^ It is active year round^7^ and feeds readily on humans and other animals during the day, particularly around dusk and dawn.^8^

Mosquito dispersal is determined by inherent species characteristics but can also be strongly influenced by environmental variables, such as openness of terrain and vertebrate host density.^9^ Container breeding species generally only travel very short distances, because they are adapted to urban environments, where blood meal sources and breeding sites are in close proximity. For example, a study of *Ae. albopictus* (Skuse) dispersal found that over 80% traveled less than 100 m, and the maximum recorded distance was 525 m.^10^ Similarly, two separate studies showed the average dispersal distance of *Ae. aegypti* (Linnaeus) to be 30.5 m under hot and dry conditions in Mexico^11^ and 78 m in tropical conditions in Cairns, Australia.^12^ In contrast, salt marsh breeding species, such as *Ae. vigilax* (Skuse) and *Ae. taeniorhynchus* (Wiedemann), can disperse large distances, so large, in fact, that direct evidence from mark–release–recapture studies is difficult to obtain because of the exponentially diminishing potential for recapture with increasing distance from the release point. However, indirect evidence of the substantial dispersal capability of these species has been shown in genetic studies of *Ae. vigilax* in Queensland^13^ and collections of *Ae. taeniorhynchus* in light traps on unmanned oil rigs up to 106 km off the coast in the Gulf of Mexico.^14^ A previous dispersal study in Victoria, Australia, on the species of interest in this paper, *Ae. camptorhynchus*, found a single individual 3 km from the release point,^15^ and similar studies in WA have shown that this species can disperse at least 4.5 km in a rural area and 6 km in an urban area.^16^ Dispersal studies have been undertaken on many other mosquito species and extensively reviewed.^9^,^17^

It is, therefore, intuitive that those people living in closer proximity to potential breeding habitat are at greater risk of mosquito-borne disease, and this relationship has been shown for malaria in Africa^18^,^19^ and Asia.^20^ Geographical information system (GIS) studies in southeast Queensland have shown that RRV rates are higher in areas with a greater proportion of native vegetation and wetlands^21^ and adult mosquito abundance.^22^,^23^ A recent study in the southwest of WA found that RRV incidence decreased with distance from a large tidal estuary with extensive mosquito breeding habitat in rural and semirural areas but found no relationship in urban areas with higher population density.^24^

The present study is the first to combine mosquito mark–release–recapture data with a GIS analysis of long-term disease data to determine the risk associated with proximity to mosquito larval habitat. It is important to quantify this risk to inform planning decisions for proposed new developments and mosquito control activities to protect existing communities near mosquito breeding habitat. The aim of this work is to investigate the mosquito-borne disease risk associated with dispersal of mosquitoes from a highly productive mosquito breeding site, known as Muddy Lakes, in the Shire of Capel, WA.

## Methods

### Setting.

**Figure 1.**
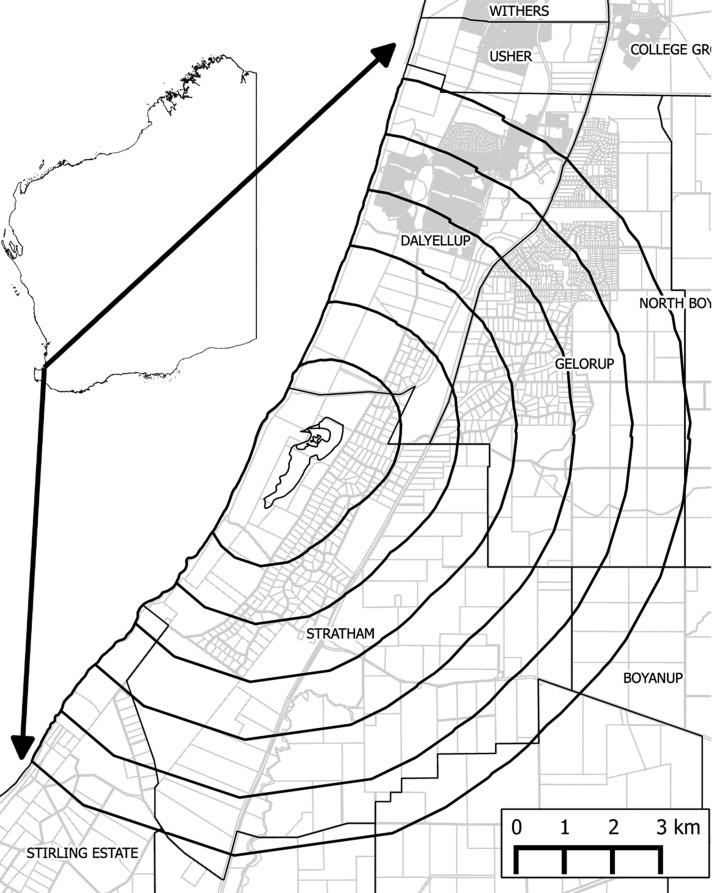
Location of Muddy Lakes with 1- to 6-km buffers.

Muddy Lakes (33°26′17.9″ S, 115°35′35.2″ E) is a wetland located in Stratham, 186 km south of Perth between Bunbury and Busselton, and it forms part of a larger 196-ha wetland system running from Harewoods Road, Dalyellup to Rich Road, Stratham (Figure 1). Stratham contains rural blocks of 2 ha or more, and neighboring suburbs include Gelorup located to the east, with block sizes from 4,000 m^2^ to 2 ha, and Dayellup, a high-density urban development to the north. All three localities are known to experience high mosquito activity at varying times throughout the year. The only recreational facilities within the area are a golf club about 2 km southeast of Muddy Lakes and a small playground 150 m behind the golf club. Substantial areas of native bushland remain in both Stratham and Gelorup, which support populations of Western Grey kangaroo (*Macropus fuliginosus*), the suspected primary natural host of RRV in southwestern Australia.^1^

Muddy Lakes are the remnants of Minninup Lakes after large drains were cut through the area to remove water that is now diverted out to sea, allowing more access to land for farming purposes. These drains are still present today, although they are not maintained. Mosquito activity in the area is relatively high and usually rainfall-driven, with large numbers of adults collected previously in the vicinity, the majority being *Ae. camptorhynchus*, and a high level of public complaints coming from surrounding areas (Shire of Capel, unpublished data). Acid sulphate soils occur naturally around Muddy Lakes^25^,^26^ and are an issue for mosquito management, because they reduce the efficacy of chemical larvicides.

Census data show that the region of interest in this study (Gelorup–Dalyellup–Stratham Statistical Area 2) grew by 242% between 2001 and 2011,^27^ and the WA Department of Planning has forecast an annual average population growth rate of 4.5% in the Shire of Capel until 2026,^28^ applying additional pressure to develop land in what is one of the highest risk areas for RRV disease in WA.

Ethics approval was not required, because our study evaluated data collected during the routine public health response to RRV as a notifiable disease.

### Dispersal study.

Many techniques have been used to study insect dispersal,^29^ and those techniques specifically relating to mosquitoes have been reviewed previously.^9^ The mark–release–recapture method was selected using a powdered pigment that fluoresced pink under ultraviolet light as the marking agent. This type of marking agent has been used successfully in many previous mosquito dispersal studies.^30^–^33^ It provides a durable, easily recognizable mark when applied in appropriate quantities and does not impact mosquito survival or behavior.^34^,^35^

Larvae were monitored at the study site so that an initial night of trapping could be conducted immediately after a new emergence to enable a large number of mosquitoes to be collected for marking and release. Additionally, recently emerged mosquitoes were more likely to survive the duration of the project and actively disperse in search of a blood meal. Encephalitis virus surveillance (EVS) light traps baited with carbon dioxide (CO_2_)^36^ and modified to suit local meteorological conditions^37^ were used to collect adult mosquitoes. Traps were placed on the western side of the wetlands along a 2,600-m transect line that ran north to south during the afternoon of October 4, 2011. They were collected the next morning and taken to the release site to be processed for release. The mosquitoes were anaesthetized by placing on dry ice for 30 seconds to 1 minute (depending on the quantity of mosquitoes in the catch bag), weighed, and placed in a large plastic bag where the fluorescent pink dust was lightly applied using a turkey baster. The mosquitoes were then placed under a tree on a tarpaulin to recover and disperse. The mosquitoes that did not recover were weighed and removed from the estimated total of mosquitoes released. The final estimated total of successfully released marked mosquitoes was 54,000.

Five recapture transect lines were identified going in north, northeast, east, southeast, and south directions from the release site, and trap sites were selected along each at distances between 300 and 6,500 m from the release point. Distances varied along each transect because of the limited availability of accessible locations to set the traps.

For the first night (October 5), recapture traps were placed at 300 and 600 m along transect A, 1,000 m along transect B, 700 m along transect C, 1,200 m along transect D, and 500 m along transect E. Another 17 traps were placed out along five transects until the study concluded on October 19. If a marked mosquito was found at the 3,500-m trapping point, another trap was placed at the 6,500-m mark. Each morning, collections were scanned using an ultraviolet (UV) light to detect any marked mosquitoes and weighed to estimate the total quantity collected. Marked mosquitoes were placed in separate specimen containers, dated, identified, and recorded. Species identification and enumeration were also carried out for all mosquitoes collected every second day.

### GIS analysis.

RRV is a notifiable disease under the Health Act (1911), requiring all cases diagnosed by a doctor or in laboratory tests to be notified to the Department of Health. Where possible, cases were followed up with a questionnaire to determine the most likely location of exposure and date of onset. If the case could not be contacted, residential address was assumed to be the location of exposure. Two time periods of RRV notification data were included in the study based on a date of onset: the most recent outbreak year from July 1, 2011 to June 30, 2012 and the 10 years from July 1, 2002 to June 30, 2012.

The dataset created for spatial analyses consisted of all cases for which data relating to place of exposure or residential address could be precisely geocoded to a specific cadastral lot (a legally defined property boundary). In addition, if place of exposure data or residential data was not given as an exact location but could be pinpointed with reasonable confidence (e.g., a street corner within 250 m), then these cases were also geocoded. All other cases were excluded from the dataset for spatial analyses.

The border of Muddy Lakes was extracted from the Hydrography Linear spatial data layer maintained by the Department of Water,^38^ and six 1-km buffers were created around this lake using Quantum GIS 1.7.4 (Open Source Geospatial Foundation, Beaverton, OR).^39^ The intersection of the buffers with the RRV case data was used to determine the number of cases within each buffer.

Property street address (PSA) cadastral data maintained by Landgate were overlaid by the Bunbury Regional Planning Scheme maintained by the Western Australian Planning Commission. Addresses in areas zoned as urban or rural were retained, and all others were excluded. Any other addresses not defined as a house were also excluded. The remaining dwellings were then used to calculate the background rate of RRV across the Shire of Capel. Finally, the PSA data were intersected with the buffers to determine the number of dwellings within each 1-km buffer.

The case and cadastral data were then summed for each buffer, the rate of RRV notifications per 1,000 dwellings was determined, and mid-P exact 95% confidence intervals were calculated. Poisson regression was then undertaken to determine if a statistically significant trend in the RRV rate with buffer distance was present. The number of RRV cases was assigned as the dependent variable, the buffer distance was the independent variable, and the number of dwellings was the exposure variable.

Finally, to determine the expected background rate, the number of RRV cases and dwellings for the whole of the Shire of Capel was calculated using the same methods described above. The rate for each buffer was determined to be significantly elevated if the 95% confidence interval did not span the background rate.

## Results

### Dispersal.

**Figure 2.**
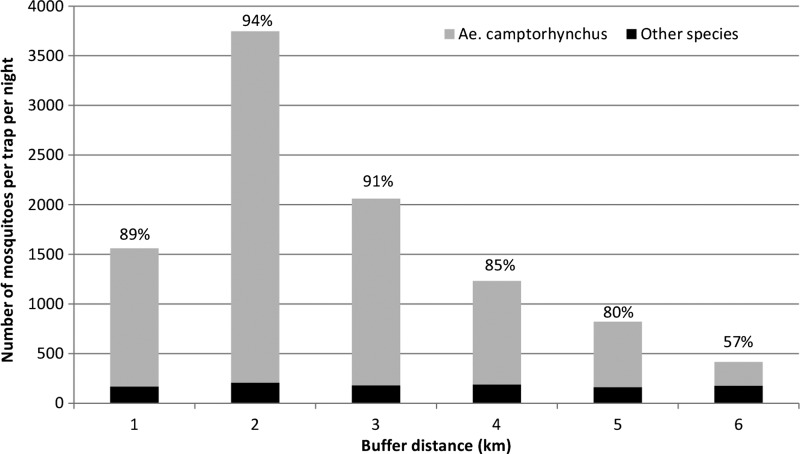
Total number of mosquitoes collected per trap per night by buffer distance during the course of the study and percentage of *Ae. camptorhynchus*.

**Figure 3.**
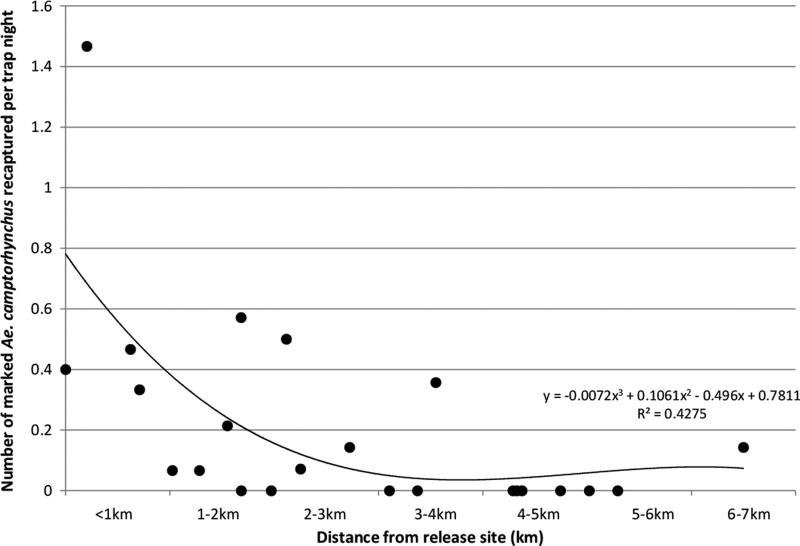
Scatter plot showing the number of marked *Ae. camptorhynchus* collected per trap night over the course of the study versus distance from the release point.

**Figure 4.**
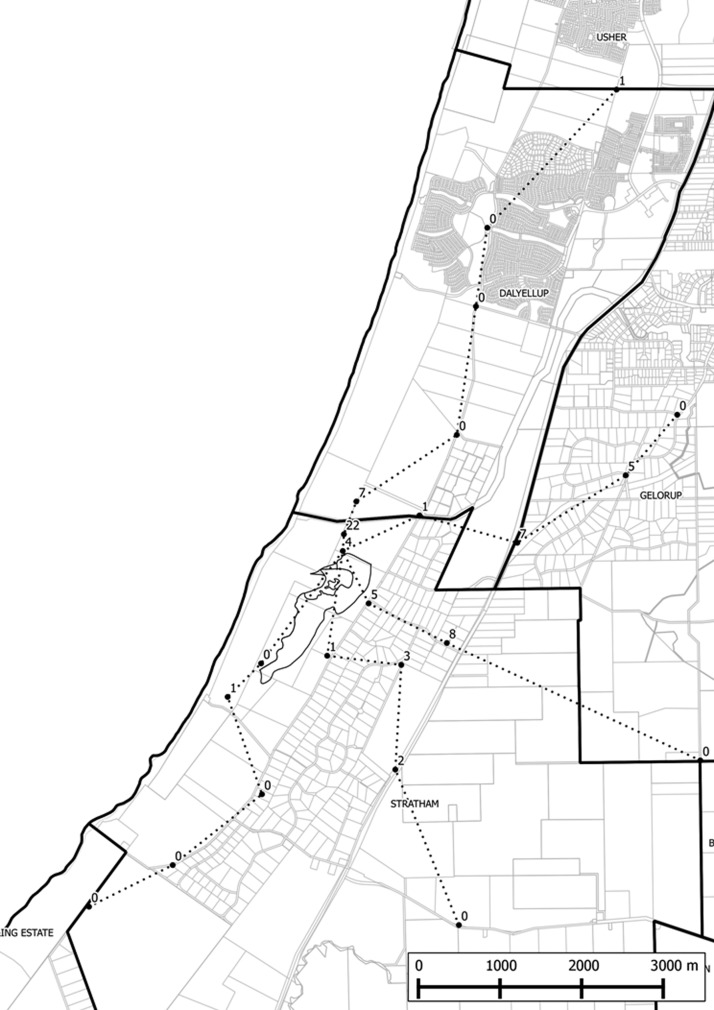
Map illustrating dispersal study trap sites and numbers of marked *Ae. camptorhynchus* recaptured.

The average number of mosquitoes collected per trap per night and the overall proportion by species over the duration of the recapture phase of the study are shown in Table 1. Figure 2

illustrates the average number of *Ae. camptorhynchus* and other species collected by buffer distance. In total, 83 marked mosquitoes were recaptured, of which 68 mosquitoes were *Ae. camptorhynchus* (82%), 10 mosquitoes were *Culex globocoxitus* Dobrotworsky (12%), 1 mosquito was *Ae. ratcliffei* Marks, 1 mosquito was *Culiseta atra* Lee, and 1 mosquito was *Cx. australicus* Dobrotworsky and Drummond. Another two mosquitoes were not able to be identified because of damage to the specimen. Figure 3

shows the number of recaptured *Ae. camptorhynchus* per trap per night versus distance from the release point overlaid by a cubic polynomial regression line, which shows a rapid decline in recaptures out to 3 km and a very low likelihood of *Ae. camptorhynchus* being recaptured beyond this distance. In fact, 91% of the *Ae. camptorhynchus* recaptured were within 3 km of the release site (Figure 3). Most (53%) were caught in north and west (14%) directions (Figure 4). The farthest recapture was 9 days after release on the opposite side of the high-density urban Dalyellup development from Muddy Lakes (6,470 m from the release site), showing that mosquito production from Muddy Lakes is impacting the surrounding residential areas.^40^

### GIS analysis.

**Figure 5.**
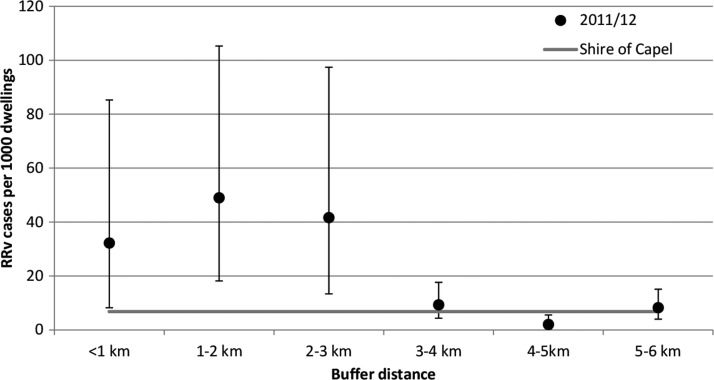
Number of RRV cases per 1,000 dwellings versus buffer distance from Muddy Lakes in 2011/2012.

The background rate of RRV per 1,000 dwellings in the Shire of Capel during 2011/2012 was 6.81. The number of cases per 1,000 dwellings in each buffer around Muddy Lakes during the 2011/2012 outbreak is shown in Table 2. A decreasing trend with increasing buffer distance is evident from 1 km on (Figure 5), and the overall decreasing trend was significant (*P* < 0.01). The number of RRV cases per 1,000 dwellings was significantly higher than the rate across the Shire of Capel for buffer distances of < 3 km during 2011/2012.

**Figure 6.**
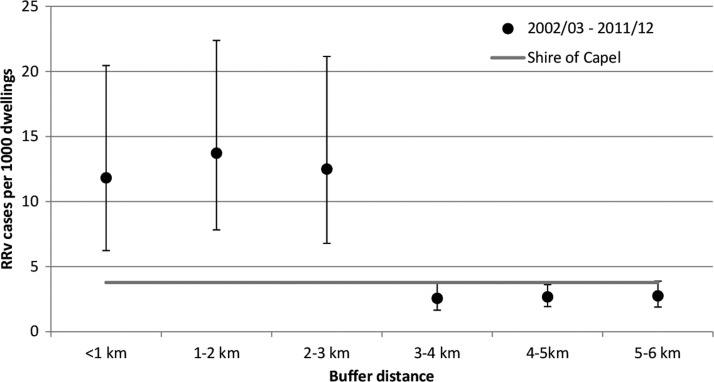
Number of RRV cases per 1,000 dwellings versus buffer distance from Muddy Lakes from July of 2002 to June of 2012.

Figure 6 shows the annual average number of RRV cases per 1,000 dwellings for each buffer distance over the 10-year period between July of 2002 and June of 2012. The rates were lower overall but again, significantly higher than the rate of 3.78 cases per 1,000 dwelling across the Shire of Capel for the first 3 km. Although the decreasing RRV rate with buffer distance was not as clear, the overall trend was still statistically significant (*P* < 0.01).

## Discussion

A significantly increased risk of contracting RRV was associated with living in close proximity to Muddy Lakes because of the presence of extensive breeding of the RRV vector, *Ae. camptorhynchus*, and marsupials that act as natural vertebrate hosts. Compared with the Shire of Capel as a whole, the 2011/2012 rate of RRV cases per 1,000 dwellings was 4.7 times higher in the < 1 km buffer, 7.2 times higher between 1 and 2 km, and 6.1 times higher between 2 and 3 km. Similarly, a significantly elevated risk was observed within 3 km of Muddy Lakes over the 10-year period between July of 2002 and June of 2012. Previous spatial analysis of RRV cases data around *Ae. camptorhynchus* salt marsh breeding habitat in the Leschenault Estuary in southwest WA also found an elevated risk of RRV out to approximately 2 km.^24^ The RRV case data are supported by the mosquito dispersal experiment, in which over 90% of the marked *Ae. camptorhynchus* were recaptured within 3 km from the release point. Furthermore, of the mosquitoes collected over the course of the study, 75% of all species and 78% of *Ae. camptorhynchus* were collected within 3 km from Muddy Lakes. The reduced RRV disease risk > 3 km from Muddy Lakes is, therefore, most likely because of the limited dispersal distance of this species and the dilution of the mosquitoes emanating from the wetland into larger areas as distance from the breeding habitat increases.

The primary limitation of this study is the accuracy of exposure location for the RRV data. As described previously, where possible, cases were followed up to determine the travel history and exposure to biting mosquitoes during the incubation period. However, this enhanced surveillance information could only be obtained for less than one-half of the cases. Where follow-up data were not available, residential address was assumed to be the location of exposure. Although this assumption means that the exposure location of some of the RRV cases included in this study was not accurate, there is no reason to suspect that the proportion of cases with inaccurate exposure information would have varied across the buffers, and therefore, the potential for differential bias to be introduced is low.

The other main limitation in this study was the need to use dwelling counts to approximate the population at risk. Properties zoned as anything other than urban or rural were removed in an effort to examine residential properties only. However, it is not possible to determine the proportion of non-residential properties that remained in the dwelling count. Furthermore, PSA data were only available for 2012; therefore, rates over 10 years are likely to be an underestimate, because fewer dwellings would have been present in previous years. Nevertheless, again, there is no evidence that these limitations in the dwelling counts were different between the buffers, and therefore, they are unlikely to significantly bias the outcomes of the study.

This study has implications for both existing and proposed developments in close proximity to wetlands. Where residential areas already exist, a detailed mosquito management plan should be developed to ensure that viable measures have been considered and can be applied to reduce the risk of exposure to virus-carrying mosquitoes among residents within 2 km of known mosquito breeding habitats. An effective mosquito management program will be based on an integrated approach that combines appropriate control measures and regular mosquito monitoring to ensure the risk of mosquito-borne disease remains at acceptable levels.

Ideally, new residential developments should not be placed or approved within 2 km of recognized permanent or semipermanent natural mosquito breeding sites, such as wetlands, salt marshes, and estuarine environments, unless exposure to mosquitoes can be permanently maintained at acceptable levels. However, reality dictates that most subdivision proposals will receive approval because of reduced land availability and the desire to live close to water bodies. Therefore, known mosquito breeding wetlands should be incorporated into land use planning scheme maps to ensure that they are accurately delineated and that the implications are considered when planning decisions are made. Notifications should be placed on the land titles within 2 km of breeding habitat, with a warning of the significantly increased health risk because of the close proximity to mosquito breeding wetlands. Some local governments in WA require developers to contribute a one-off fee to the cost of ongoing mosquito control by the local authority for developments in known high-risk areas, which could also be considered for future developments in this location.

## Figures and Tables

**Table 1:** Average number and proportion of mosquitoes collected per trap per night during the study

Species	Average/trap/night	%
*Ae. alboannulatus*	3.4	0.2
*Ae. camptorhynchus*	1,716.5	89.9
*Ae. clelandi*	0.6	< 0.1
*Ae. notoscriptus*	0.5	< 0.1
*Ae. ratcliffei*	22.9	1.2
*Anopheles annulipes*	3.4	0.2
*Cx. australicus*	59.5	3.1
*Cx. globocoxitus*	101.2	5.3
*Coquillettidia* species near *linealis*	0.5	< 0.1
*Culiseta atra*	1.4	0.1

**Table 2:** Number of dwellings, RRV cases, and rate per 1,000 dwellings by buffer distance from Muddy Lakes in 2011/2012 and from July of 2002 to June of 2012

Buffer distance (km)	Dwellings	2011/2012		From July of 2002 to June of 2012	
		Cases	Average annual cases/1,000 dwellings	Cases	Average annual cases/1,000 dwellings
< 1	93	3	32.26	11	11.82
1–2	102	5	49.02	14	13.72
2–3	96	4	41.67	12	12.50
3–4	856	8	9.35	22	2.57
4–5	1,455	3	2.06	39	2.68
5–6	1,087	9	8.28	30	2.76
